# Comment on “Optimal Nutritional Status for a Well-Functioning Immune System Is an Important Factor to Protect against Viral Infections. *Nutrients* 2020, *12*, 1181”

**DOI:** 10.3390/nu12082321

**Published:** 2020-08-02

**Authors:** Alexandros Tsoupras, Ioannis Zabetakis

**Affiliations:** 1 Department of Biological Sciences, University of Limerick, V94 T9PX Limerick, Ireland; alexandros.tsoupras@ul.ie; 2 Health Research Institute, University of Limerick, V94 T9PX Limerick, Ireland; 3 Bernal Institute, University of Limerick, V94 T9PX Limerick, Ireland

The novel coronavirus (COVID-19) pandemic, caused by severe acute respiratory syndrome coronavirus 2 (SARS-CoV-2), has engulfed the world since December 2019. Since then, many studies have focused on the impact that lifestyle and dietary intake of nutrients have on immune system and respiratory tract infections. The supplementation of several nutrients, including omega-3 polyunsaturated fatty acids (n-3 PUFA), such as eicosapentaenoic (EPA) and docosahexaenoic (DHA) acids, has recently been proposed as a support towards optimal immune function [1]. The authors of the aforementioned article proposed a daily intake of 250 mg EPA and DHA, based on recommendations for optimal immune function, according to global, regional and national guidelines [1]. 

n-3 PUFA came into attention since the discovery that Greenlandic Inuits had a lower risk of developing cardiovascular diseases (CVD) due to their fish-rich diet. However, the observed beneficial effects of fish and fish oils are likely to be mediated through the interplay of a plethora of lipids, rather than just the neutral forms (i.e. free fatty acids or esters) of n-3 PUFA [2,3]. In addition, in several systematic reviews and meta-analyses, inconsistent and mixed outcomes in relation to the potential benefits of these neutral forms of n-3 PUFA against inflammation-related pathologies, including CVD, have been reported [2,4,5,6,7].

Moreover, recent atherosclerotic CVD trials have not demonstrated any significant cardiovascular benefit in patients taking 1 g per day of EPA and DHA as supplements in conjunction with their standard CVD treatment [8,9]. We also need to highlight that only high doses (>4 g/day) of the esters of EPA and/or DHA may elicit a cardiovascular benefit, as shown in the REDUCE-IT trial [10]. 

All these recent evidences do not support the view that low doses of 250 mg EPA and DHA mentioned by Calder et al. [1] have any beneficial effect on immune function against respiratory infections, or in general against inflammation and related disorders such as CVD. It should also be noted that in the very same guidelines that Calder et al [1] used for the recommendation of 250 mg EPA and DHA (EFSA position in reference 81 of Calder et al. [1]), it is concluded, as in other articles, that a dose exceeding >1 g and up to 2 g of n-3 PUFA is required to observe any effect on inflammation. 

Therefore, in the midst of this severe COVID-19 pandemic, we would wish to emphasize, as a matter of urgency, the appropriate use/citation of the existing guidelines by all, but also a call to the competent authorities (EFSA, FAO, Chinese Nutrition Society) to re-validate and re-evaluate their possibly out-of-date recommendations-guidelines on n-3 PUFA intake (as esters), in relation to their dose-efficacy and safety, for promoting public health. Higher safe dose recommendations are required for immune and CVD function.
